# Is there a link between Depressive Disorders and Tryptophan Hydroxylase 1 (TPH1) Gene Polymorphism? - Study from a Distressed Area, Kashmir (India)

**DOI:** 10.7759/cureus.673

**Published:** 2016-07-06

**Authors:** Raheel Mushtaq, Shah Faisal Ahmad Tarfarosh, Mohammad Maqbool Dar, Arshad Hussain, Sheikh Shoib, Tabindah Shah, Sahil Shah, Mushbiq Manzoor

**Affiliations:** 1 Mood Disorder Clinic, Postgraduate Department of Psychiatry, Government Medical College, Srinagar, J & K, India; 2 MBBS, Acharya Shri Chander College of Medical Sciences and Hospital, Jammu, J & K, India; 3 Postgraduate Department of Psychiatry, Government Medical College, Srinagar, J & K, India; 4 MBBS, Government Medical College, Srinagar, J & K, India; 5 MBBS, Acharya Shri Chander College of Medical Sciences and Hospital, Sidhra, J & K, India; 6 MBBS, Sheri Kashmir Institute of Medical Sciences Medical College, Srinagar, India

**Keywords:** tryptophan hydroxylase 1, tph1, mdd, polymerase chain reaction, genetics, neurology, neuroscience, gene polymorphism, brain networks, behaviour neurology

## Abstract

**Background:**

The progress that man has made in all domains of life, during all these years of reign over the earth, is utterly remarkable. However, it always came at a price. Each epoch of progress has seen human beings inflicted with trauma and cynical consequences. During the last two decades, Kashmiri (Indian) people have experienced continuous violence, a reign of terror, and political turmoil. Each of these disastrous events has contributed to the increase in psychiatric disorders in this part of the world, especially major depressive disorders. We can observe that besides the environmental influences, gene polymorphism also plays a crucial role in the development of depressive disorders. The role of Tryptophan Hydroxylase 1 (TPH1) gene is implicated in various psychiatric disorders, including depression. However, no study has investigated TPH1 A779C gene polymorphism in depressive disorders in a distressed society like Kashmir (India).

**Aims:**

To study TPH1 A779C single nucleotide polymorphism in depressive disorders in Kashmiri (Indian) population.

**Materials and Methods:**

Two hundred and forty patients diagnosed with depressive disorder, and 160 unrelated healthy volunteers (control), were studied in a case-control study design. Polymorphism was determined using polymerase chain reaction (PCR) and agarose gel electrophoresis, after digestion with HAP II enzyme. Genotypes and allele frequencies were compared using Chi-square tests, Fisher’s exact test, odds ratio, 95% confidence interval (C.I.) and a p-value of <0.05 was considered to be statistically significant.

**Results:**

The mean age ± standard deviation (SD) of depression and control group was 32.02±10.99 and 31.75±9.93, respectively (p= 0.512). It was found that the patients from depression group had AA genotype (51.7%) in comparison to control group (17.5%) and these results were statistically significant (p≤0.0001). Calculation of allelic frequency revealed a stronger association of A allele with depression group (70.83%) than with the control group (41.25%), and it was also found to be statistically significant (p≤0.0001) with C.I. of 3.459 (1.909-6.266).

**Conclusion:**

TPH1 A779C A gene was found to be associated with a major depressive disorder (MDD) in Kashmiri (Indian) population. There were high HAM-A as well as HAM-D scores in depressive patients of Kashmir (India).

## Introduction

Trauma is an inevitable component of human existence in a conflict area. The last two decades in Kashmir (India) have seen around 20,000 deaths and 4,000 disappearances. There have been episodes of continuous violence, political turmoil, and reign of terror, all of which have increased the overall psychiatric morbidity [1-2]. Depression is one of the many psychiatric disorders that can occur due to traumatic events. It is one of the most common types of psychiatric disorders and a worldwide health problem, especially in a developing country like India. The point prevalence of unipolar depressive disorders in the world is 1.9 % in males and 3.2 % in females. Moreover, a one-year prevalence rate of unipolar depression is 5.8 % in males and 9.5 % in females [3]. The estimated prevalence of depression in India, in the community, is estimated to be from 1.7 to 74 per thousand populations. Depression is estimated to become the second leading cause of disability, after ischemic heart disease [3].

Major depressive disorders (MDD) result from a complex interaction between an individual’s genetic endowment and environmental factors. The influence of environment is difficult to evaluate in a genetic association study and is usually a confounding factor [1, 2]. In recent years, attempts to investigate genetic influence for the development of depressive disorders have emerged as one of the prominent areas of neuroscience research [4]. Various candidate genes have been studied for possible association with a major depressive disorder (MDD) [1-3]. Several investigators have pointed out that TPH (Tryptophan hydroxylase) gene may be associated with some psychiatric disorders. Considerable evidence has shown that the TPH gene, which encodes for the enzyme tryptophan hydroxylase, is a possible candidate involved in the etiology of major depressive disorder (MDD) [3, 5, 6].

Tryptophan hydroxylase (TPH) is a rate-limiting enzyme involved in the biosynthesis of the serotonin [2]. TPH gene has two isoforms, i.e., TPH1 and TPH2 [7, 8]. TPH1 gene is mapped on chromosome 11p15.3-p14. It is about 29 kb long and contains 11 exons [5, 9]. Tryptophan hydroxylase 1 protein is present in the gut, spleen, thymus, pineal and pituitary gland [10]. On the other hand, TPH2 is predominantly expressed in the brain stem, where the serotonergic raphe nuclei are located [4, 5].

There are various variants of TPH1 referred to in literature. Different studies have shown an association of TPH1 gene variants with depressive disorders [5, 7, 9]. Previously, in our earlier study, we found no relationship of another isoform of TPH gene (TPH2) in depressive disorders in Kashmiri (Indian) population [1, 2]. As the association of TPH1 gene in depressive disorders in some studies has shown positive association and it was not possible to study all the variants of TPH 1 gene, due to financial and time restraints, TPH1 A779C variant was selected. However, all those studies showing a positive association of TPH1 A779C gene with depressive disorders were done in western countries [9, 11]. No study of this variant of the gene has been done in India. In the present study, we use a case-control study design to investigate the association of TPH1 A779C gene polymorphism in depressive disorders in Kashmiri (Indian) population.

## Materials and methods

### Setting

The study was conducted in Mood disorder clinic, Institute of Mental Health and Neuro-Sciences (IMHANS) associated with Government Medical College, Srinagar, Kashmir (India), which is the lone tertiary psychiatric hospital in Kashmir and caters to the majority of psychiatric patients of Kashmir [2, 3].

### Sample size

A total of 400 unrelated individuals (240 with unipolar major depression and 160 healthy volunteers) were enrolled in the study from July 2010 to August 2014 in our institute. One hundred and sixty unrelated healthy volunteers belonging to the same state were selected (from University of Kashmir), after excluding mental disorders by a comprehensive clinical interview by two experienced psychiatrists.

### MDD diagnosis

The diagnoses of major depression were made based on Diagnostic and Statistical Manual of Mental Disorder (DSM IV TR) criteria [12]. Bipolarity was excluded by complete history taking and detailed mental status examination. Diagnoses were confirmed by two consultant psychiatrists independently.

### Inclusion criteria

1.  Persons suffering from depressive disorders.

2. Patients above 18 years of age.

3. Patients willing to participate in the study, and were asked to sign up for informed consent.

### Exclusion criteria

1. Patients below 18 years of age.

2. All depressive disorders due to general medical conditions and due to psychoactive substances use; exclusion was done before selection of patients.

### Measurements

Observer rating scales like Hamilton anxiety rating scale (HAM-A) and Hamilton rating scale for depression (HAM-D) were administered to the study participants. Both the scales have good reliability and validity [13, 14].

HAM-A is a 14 item scale, which is used to assess the severity of anxiety. A score of 17 or less indicates mild anxiety and a score of 18 to 24 indicates moderate anxiety. A score above 25 indicates severe anxiety [13].

HAM-D is a 17 item scale used to assess the severity of depression. It has good validity and reliability. A score of 0 to 7 is considered to be normal. A score of 20 or above indicates moderate or severe depression [14].

### DNA extraction

For genotyping, the DNA was extracted from a portion of whole blood, using GENEI Genomic Extraction Kit, supplied by the Messers Bangalore Genei, India.

### Determination of TPH1 A779C gene polymorphism

Polymerase chain reaction was carried out with primer (5- ATGTGTGAAAGCCTTTGACCCAAAG ACA) and Reverse (5- TGCGTTATATGACATTGACTGAACT GC) [5, 2]. PCR was performed in 20 ul mixture containing about 50 ng genome DNA, ten pmol of each primer, Tris-HCL, pH=8, 100 uMdNTP’s, 1U of Taq Polymerase using Tech-gene–Thermal cycler (UK). The amplification was carried out in the following way: after an initial incubation at 95 degree Celsius for 10 minutes, 30 cycles of denaturation at 94 degree Celsius for 1 minute, an annealing step at 65 degree Celsius for 1 minute, followed by an extension step at 72 degree Celsius for 1 minute were performed and final incubation at 72 degree Celsius for 10 minutes was done. After a 3% agarose gel electrophoresis, the PCR products were stained with ethidium bromide and then bands were observed under UV light.

The allelic size was determined by the comparison of bands with size standards after electrophoresis in polyacramide gel, followed by silver staining, and three genotypes i.e. GG, AA and AG were observed. Fragment A had 468 base pairs and G had two bands of 244 and 224 bp. Randomised selected DNA samples were subjected to direct sequencing to validate the genotype.

**Figure 1:**
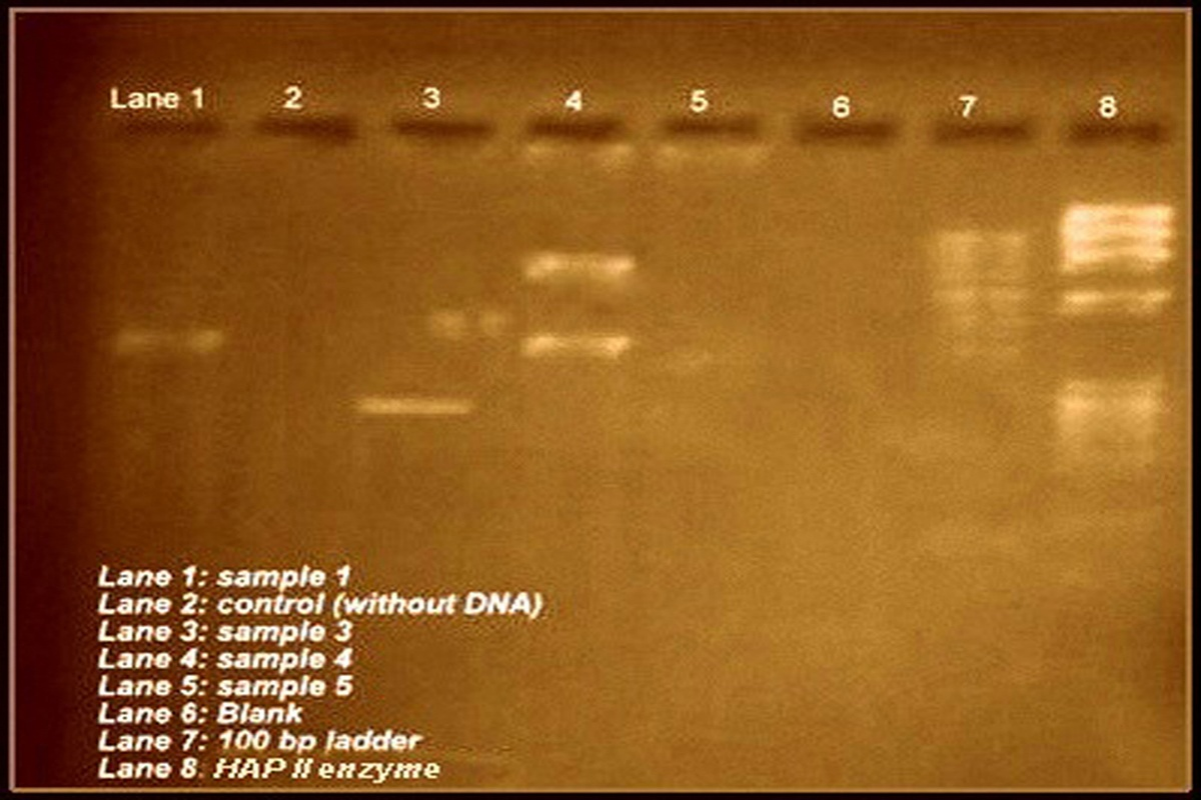
PCR amplification of TPH1 gene Agarose gel electrophoresis of TPH1 A779C gene, using HAP II endonuclease enzyme

PCR amplified fragments were digested with Hap II restrictive endonuclease and were analysed by electrophoresis on 1.5% Polyacramide gel. Sequencing was done subsequently, using an automatic sequencer – three genotypes CC-AC-AA were observed as shown in Figure 1.

### Statistical analysis

Genotype and allele frequencies were compared using Chi-square test, Fisher's exact test, odds ratio, 95% confidence interval (C.I.) and a p-value of < 0.05 was considered to be significant. The quantitative data was analysed by one-way analysis of variance (ANOVA) and two sample independent t-tests. All data were analysed using SPSS version 13 for Windows (IBM SPSS Statistics).

### Consent and approval

The study was done after obtaining clearance from the ethical committee of the GMC, Srinagar, India, and no grant was funded by the committee. The patients and volunteers gave their consent before being subjected to various tests.

## Results

The mean age ± SD of depression and control group was 32.02 ± 10.99 and 31.75 ± 9.93, respectively, and age difference between the groups was found to be statistically non-significant (p = 0.512). As shown in Table 1, the mean HAM-A in depression group and control group was 16.52 and 6.88, respectively, and the difference was found to be statistically significant (p ≤ 0.0001). The mean HAM-D in depression and control group was 25.2 and 5.75, respectively, and this difference was also found to be statistically significant (p ≤ 0.0001).

**Table 1:** Mean age and Results: HAM-A and HAM-D in studied groups SD = Standard deviation HAM-A = Hamilton Anxiety Rating Scale HAM-D = Hamilton Depression Rating Scale Values within parenthesis are percentages * Significant at p < 0.05

	MDD n=240	Control n=160	p-value
Age (Mean ± SD)	32.02(10.99)	31.75 (9.93)	0.512
Male	35(58.3 )	25(62.5)	
Female	25(41.7)	15(37.5)	
HAM-A	16.52(5.61)	6.88(2.49)	≤0.0001^*^
HAM-D	25.2(5.58)	5.75(2.45)	≤0.0001^*^

The two groups of patients were compared on the basis of genotypic and allelic frequency as shown in Table 2. It was found that patients from depressive group had AA genotype (51.7%) compared to controls (17.5%) and it was found to be statistically significant (p ≤ 0.0001). Comparison of allelic frequency revealed association of A allele in depression group (70.83%), compared with control group (41.25%) and it was also found to be statistically significant (p ≤ 0.0001), with C.I. of 3.459 (1.909 - 6.266).

**Table 2:** Genotypic and allelic frequency of gene in the studied group (TPH1 A779C) O.R. = Odds ratio C.I. = Confidence Interval Values within parenthesis are percentages * Significant at p < 0.05

Genotypes						Allelic frequency			
Group	AA	AC	CC	Chi- square test	p-value	A	C	p-value	O.R. (95% C.I.)
Depressive n=240	124(51.7)	92(38.3)	24(10)	15.35	≤0.0001^*^	340(70.83)	140(29.16)	≤0.0001^* ^	3.459 (1.909 - 6.266)
Control n=160	28(17.5)	76(47.5)	56(35)			132(41.25)	188(58.75)		

## Discussion

Since time immemorial, the valley of Kashmir has been regarded as the paradise on earth because of its splendid natural beauty. Over the last eighteen years, Kashmir valley has seen many downfalls, due to continuous violence, political turmoil, and reign of terror [1, 2, 15]. The last two decades have seen a rise of psychological and psychiatric disorders in Kashmir [2, 3]. There have been around 20,000 deaths and 4,000 disappearances in Kashmir (India) over the last 20 years. The lifetime prevalence of any trauma in Kashmir in 2006 was 59.50 percent for males and 57.39 percent for females [16, 17]. The increase of depressive disorders in Kashmir (India) is primarily due to continual conflict, elevated stress in daily life and genetic factors. It is well known that genetic factors contribute approximately 40% towards the risk of major depressive disorders [2].

To the best of our knowledge, this is the first study which investigates the association of TPH1 A779C with depressive disorders in Kashmiri (Indian) population. Though there was no deliberate effort to match the control and patient groups for age, both of them were found to be matched for age. Further, the mean anxiety and depressive scores (HAM-A and HAM-D) were higher in depression group compared to controls. A difference in the mean scores was found to be statistically significant (p ≤ 0.0001). Anxiety symptoms commonly occur in patients with depressive disorders. Increased symptoms of anxiety in depressive patients can be explained by shared genetic vulnerability to both the disorders. Studies have shown that TPH1 gene polymorphism is also related to the anxiety symptoms in MDD [4, 18].

In the present study, we found an association of TPH1 A779C polymorphisms with MDD in Kashmiri (Indian) population. Our findings suggest that AA genotype (50%) and the frequency of A allele (70.28%) in TPH1 A779C was found associated with major depressive disorders (p ≤ 0.0001). However, our findings are in contrary to a few studies [19, 20] and at the same time, the results of our findings are by several studies [21, 22, 23]. In a study done by LH Lian et al. (2013) in three ethnic groups of Malaysia (Malaysians, Chinese and Indian), no association of TPH1 gene was found with MDD. However, in the same study, haplotype analysis suggested that in the Indian population, TPH1 might be a risk factor for MDD [24].

In our earlier study on TPH2 gene polymorphism, in Kashmiri population, there was no association of AA genotype and A allele frequency in the depression group. Although the percentage of AA genotype (72.41%) and the frequency of A allele (75.55%) were high, the difference was not found to be statistically non-significant ( p = 0.460, OR p = 1.25 (0.69 to 2.25)).The variability in the results of various studies can be explained by ethnic variation [2]. In the same population, cultural and environment factors could explain differences in the genetic components [2, 4].

It is not known with certainty that which of the alleles are the risk-alleles (A or C allele) for predisposition of MDD and this is still a topic of debate. In our study, an excess of A allele (70.28%) was found in the depressive patients of the Kashmiri (Indian) population. It is also known that the 779A allele is found more in the Indian population. Moreover, Jokela et al. found an increase in depressive features in patients having A allele [24]. Other studies have also shown that A allele might have a significant impact on depressive disorders [24, 25]. However, some authors are of the view that A allele might be a protective factor against depressive symptoms in MDD patients [24, 25].

Our study is first of its kind conducted in the North-Indian subcontinent mountainous valley of Kashmir, where susceptibility towards MDD is high, due to ongoing conflict in the area, thus, further magnifying the effects of genetic predisposition. Further, large samples are needed to study and understand the role of these genetic variants on depressive disorders.

### Limitations

1. The study was conducted in one big centre only and, therefore, the generalization of the results may be questioned.

2. Another limitation included the possibility of selection bias as the sample was drawn from the hospital only.

## Conclusions

Although genetics of depressive disorders appears to be understudied and least explored in India, our preliminary study shows that TPH1 A779C was found associated with MDD in Kashmiri population. There were high HAM-A as well as HAM-D scores in depressive patients of Kashmir (India).
